# Diagnosing and Managing Trophoblastic Lesions in Cesarean Scars: A Systematic Review

**DOI:** 10.7759/cureus.86647

**Published:** 2025-06-24

**Authors:** Asma Malik Sadaqat, Umme Habiba, Mehwish Khan, Zareena Begum, Muhammad R Waris, Hameed Ullah, Maheen Zahid

**Affiliations:** 1 Department of Obstetrics and Gynaecology, District Health Authority, Rawalpindi, PAK; 2 Department of Obstetrics and Gynaecology, Valley Clinic, Rawalpindi, PAK; 3 Department of Obstetrics and Gynaecology, Clinica Medical Centre, Islamabad, PAK; 4 Department of Obstetrics and Gynaecology, Saidu Teaching Hospital, Swat, PAK; 5 Department of Emergency Medicine, Carnival Cruise Line, Florida, USA; 6 Department of Paediatrics, Lady Reading Hospital, Peshawar, PAK; 7 Department of Obstetrics and Gynaecology, King Edward Medical University, Lahore, PAK

**Keywords:** cesarean scar ectopic pregnancy, choriocarcinoma, ectopic molar pregnancy, ectopic pregnancy, gestational trophoblastic disease, hydatiform mole

## Abstract

Gestational trophoblastic disease (GTD) arises from an aberrant placenta and includes a spectrum of disorders ranging from premalignant to malignant. Changes in the epidemiology of GTD have been noted in various countries. Misdiagnosis can result in serious complications either because of the natural course of the disease or because of inadequate therapy that ensues. A systematic literature search was conducted in May 2025 using PubMed, Google Scholar, and the Cochrane Library. MeSH terms for "trophoblastic lesion" and "cesarean scar" were applied. Case reports describing trophoblastic disease in cesarean scars in women of any age were included. Non-trophoblastic lesions, cases not in cesarean scars, and non-English articles were excluded. Data were extracted from included studies and assessed using the Joanna Briggs Institute (JBI) Critical Appraisal Checklist for Case Reports. The review followed PRISMA guidelines. Thirty-six case reports from 2006 to 2024 were included. Patients ranged from 22 to 54 years and were multiparous with prior cesarean sections. Common symptoms included vaginal bleeding, amenorrhea, and pelvic pain. Serum β-hCG levels varied, with elevated levels in choriocarcinoma (CC) and normal or low levels in atypical placental site nodules (APSNs) and epithelioid trophoblastic tumors (ETTs). ETT was the most frequent lesion type, followed by CC and APSNs. Diagnosis used transvaginal ultrasonography (TVUS), Doppler imaging, and magnetic resonance imaging (MRI), confirmed by histopathology. Treatment was mainly surgical; chemotherapy was used in CC cases. Outcomes were favorable, with no recurrence in follow-up. Trophoblastic lesions in cesarean scars require heightened clinical awareness due to their diagnostic complexity. Early identification using imaging and histopathology is crucial. This review emphasizes standardized diagnostic pathways and the need for studies on management protocols.

## Introduction and background

Gestational trophoblastic disease (GTD) is a group of cancerous disorders that originate in the placenta. While placental site trophoblastic tumor (PSTT), epithelioid trophoblastic tumor (ETT), gestational choriocarcinoma (CC), and hydatidiform moles are histologic diagnoses, postmolar gestational trophoblastic neoplasia (GTN) is diagnosed by clinical and laboratory criteria. While the disease entities that fall under GTD exhibit a wide range of behaviors, GTN refers only to those that can invade tissues and metastasize [1]. The recognition of GTD dates back to ancient times. Sometimes, individual hydropic molar villi are seen as distinct fetuses. Velpeau and Boivin identified hydatidiform moles as cystic dilatation of the chorionic villi around the beginning of the 1800s [2]. Molar pregnancy, a kind of GTD, occurs when 0.6-8 out of every 1,000 pregnancies are caused by aberrant trophoblasts that have the potential to develop into cancer [3]. The disorder affects both partial and complete moles, two genetically distinct but closely related forms of abnormal pregnancies. Although the probability of malignant transformation is considerably higher for complete moles, both have the potential [4].

Cesarean scar pregnancy (CSP) is a late, serious complication of a cesarean section, characterized by an ectopic pregnancy [5] implanted in the myometrium of a prior cesarean scar [6]. Among women who have had at least one previous cesarean section and an ectopic pregnancy, the incidence of CSP is 1:2,216, while its rate is 6.1% [7]. CSP has a very low incidence, but with the increase in the number of cesarean sections recently, it has been growing. It is believed to result from defective healing of the myometrium and endometrium at the incision site. Inadequate myometrial regeneration or fibrotic healing leads to a microscopic dehiscent tract or wedge-shaped defect, which can create a pathway for the blastocyst to implant abnormally. Once implantation occurs, the poorly vascularized scar tissue may fail to provide adequate support, increasing the risk of abnormal placentation, including placenta accreta spectrum disorders and GTD [7]. Although the mortality and morbidity rates have significantly decreased over the last two decades due to early detection, appropriate blood transfusion, and infection management, ectopic pregnancy with GTD is rare [8]. Successful CSP treatment requires early diagnosis and treatment, which depends on color Doppler ultrasonography. Notably, misdiagnosis is common because cesarean scar CC frequently mimics CSP [9]. Treatment delays, unsuccessful treatments, or metastases may result from this misclassification [10]. Marchand reported in 1895 that CC develops after a hydatidiform mole and less often during normal pregnancy [2]. Therefore, early detection and timely lesion removal are essential components of CC management [11].

There is no consensus on the optimal approach and criteria for diagnosing this uncommon disease. Quantitative measurement of beta-human chorionic gonadotropin (β-hCG) is a fundamental diagnostic tool in the evaluation of abnormal pregnancies. In CSP and GTD, serum β-hCG levels may exhibit atypical patterns. For instance, in CSP, levels may rise more slowly than in normal intrauterine pregnancies or plateau in cases with arrested development [12]. Serial monitoring helps not only in diagnosis but also in assessing treatment response and disease regression or recurrence [13]. A non-invasive, low-cost medical imaging technique for diagnosing cesarean scar GTD is transvaginal ultrasonography (TVUS). Ultrasound (USG) has high sensitivity for detecting GTD. Characteristic appearances include a "snowstorm" look, a "Swiss cheese" look, or localized heterogeneous myometrial echogenic lesions with fluid. TVUS is highly effective in identifying cesarean hysterotomy scars [14]. When the gestational sac is situated in the anterior isthmic section of the uterus, corresponding to the site of a previous cesarean scar, and there is significant peritrophoblastic flow with both the uterus and cervical canal remaining empty, CSP should be considered [7]. In cases of GTD, an unusual echo pattern in the anterior isthmic region may suggest cesarean scar GTD. Furthermore, some researchers have identified three-dimensional power Doppler imaging as a valuable tool that may offer significant insights into differentiating the neovascularization characteristics associated with cervical pregnancy [15,16]. To date, many instances of CSP have been diagnosed through TVUS in the early phases of pregnancy. Michael and colleagues [17] reported that USG has an 84.6% sensitivity rate for detecting CSP. The following criteria are essential to meet in order to diagnose this condition [18,19]: (1) a gestational sac anteriorly at the level of the internal os covering the visible or suspected site of the previous cesarean section scar; (2) an empty uterine cavity; (3) a region of increased peritrophoblastic or periplacental vascularity on color Doppler examination; and (4) a negative "sliding organs sign," which is the inability to move the gestational sac from its position at the level of the internal os using light pressure from the transvaginal probe. Despite advancements in imaging technology, CSP is sometimes overlooked or misidentified as a cervical ectopic pregnancy, an aberrant intrauterine pregnancy such as trophoblastic disease or miscarriage, or a normal intrauterine pregnancy [20]. Additional imaging techniques, such as chest X-rays, computed tomography (CT) scans of the chest and abdomen, and brain magnetic resonance imaging (MRI), are employed to identify metastasis. Pelvic MRI is also beneficial for detecting myometrial invasion. GTD pathology is characterized by abnormal trophoblast proliferation. Monitoring serum β-hCG levels is crucial for diagnosing cesarean scar GTN [19].

Trophoblastic lesions of cesarean scars are a rare but clinically significant consequence of prior cesarean operations. Their early and accurate diagnosis is challenging since they can mimic other intrauterine or ectopic pathologies and may be accompanied by non-characteristic symptoms. The need for greater clinical awareness is highlighted by the fact that such aberrant implantations are anticipated to rise due to the increasing rate of cesarean sections worldwide. Despite an increase in case-based reporting in the literature, there is a lack of consensus on the most effective diagnostic pathways and standardized management techniques. In order to support clinicians in early detection and management and to identify areas that need further research, this systematic review aims to summarize the body of knowledge regarding the diagnostic modalities, clinical presentation, and therapeutic approach for trophoblastic lesions of cesarean scars.

## Review

Materials and methods

This review was conducted in accordance with the Preferred Reporting Items for Systematic Reviews and Meta-Analyses (PRISMA) 2020 guidelines.

Search Strategy

A comprehensive literature review was performed using three electronic databases, PubMed, Google Scholar, and the Cochrane Library, to locate pertinent case reports of trophoblastic lesions in cesarean section scars. The search utilized terms such as "trophoblastic tumor", "gestational trophoblastic disease", "trophoblastic lesion", "invasive mole", "choriocarcinoma", "cesarean section", and "cesarean scar", combined with Boolean operators AND and OR.

Inclusion and Exclusion Criteria

Case reports that detailed trophoblastic lesions originating in or affecting a previous cesarean section scar in women of any age were considered. The lesions included CC, ETT, PSTT, invasive mole, and atypical trophoblastic proliferation. Reports were not included if the lesion was found in a different location (such as inside the uterus but not in the scar), if they were about non-trophoblastic issues in the cesarean scar (such as fibroids, adenomyosis, scar dehiscence, or endometriosis), or if the full text was not available in English (Table 1).

**Table 1:** Inclusion and exclusion criteria

Category	Inclusion Criteria	Exclusion Criteria
Population	Women of any age	–
Condition	Trophoblastic lesions involving or originating in a previous cesarean section scar	Lesions not located in or affecting the cesarean scar (e.g., intrauterine, cervical, or other locations)
Lesion types	Choriocarcinoma; epithelioid trophoblastic tumor (ETT); placental site trophoblastic tumor (PSTT); invasive mole; atypical trophoblastic proliferation	Non-trophoblastic lesions in cesarean scars, such as fibroids, adenomyosis, scar dehiscence, and endometriosis
Study type	Case reports or case series with sufficient clinical detail	Reviews, editorials, animal/in vitro studies, or reports lacking adequate clinical information
Language	Full-text articles available in English	Articles not available in English

Study Selection and Screening

**Figure 1:**
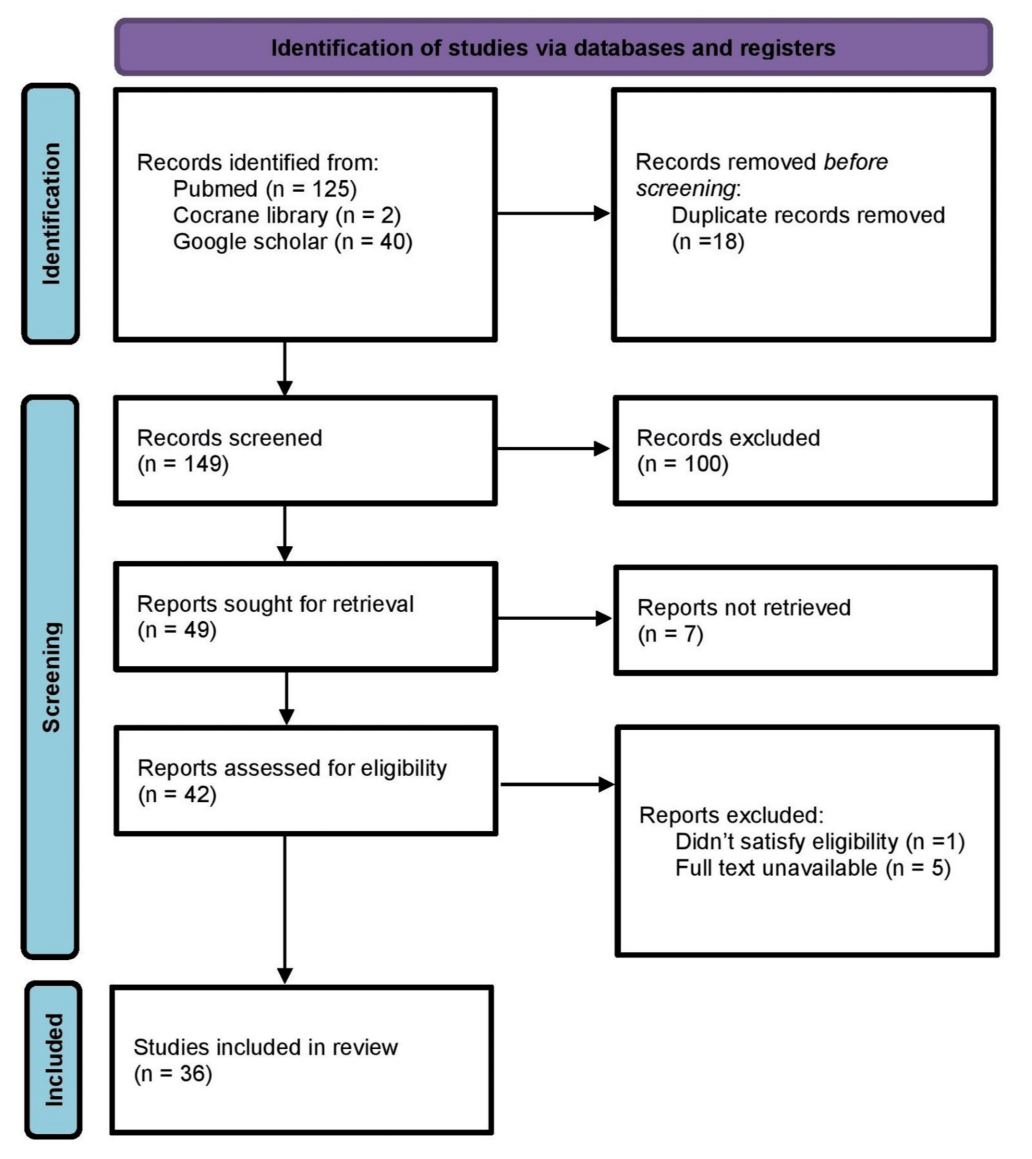
Preferred Reporting Items for Systematic Reviews and Meta-Analyses Flowchart.

All identified citations were imported into EndNote (Clarivate, London, United Kingdom), and duplicates were eliminated. Two independent reviewers assessed titles and abstracts against the inclusion criteria. Full texts of studies that appeared eligible were then examined. Disagreements were settled through discussion or with assistance from a third reviewer. The process of screening and selection followed the guidelines of PRISMA, and a PRISMA flowchart was developed to illustrate the procedure (Figure 1).

Data Extraction and Quality Assessment

Data extraction was independently carried out using a standardized tool. Information extracted included study characteristics (e.g., year, country, design), patient characteristics (age), clinical presentation, β-hCG levels (mIU/mL), diagnosis, type of trophoblastic lesion, diagnostic methods, treatment, and outcomes. Discrepancies were resolved within the research team. The quality assessment employed the Joanna Briggs Institute (JBI) Critical Appraisal Checklist for Case Reports. Each report was independently evaluated, and only those deemed high quality based on the clarity of patient history, diagnostic precision, intervention description, and outcome reporting were included in the final analysis.

Statistical Analysis

As this systematic review included only case reports on gestational trophoblastic disease in cesarean scars, a quantitative meta-analysis was not feasible due to the heterogeneity of data and lack of standardized outcome measures. Therefore, a narrative synthesis was performed, summarizing clinical presentations, diagnostic methods, management approaches, and patient outcomes.

Results

Between 2006 and 2024, 36 case reports from various geographical locations, including Taiwan, Serbia, China, and the United States, were included in this systematic review. Most of the cases involved women between the ages of 22 and 54. Lesion formation was observed months to years after index surgery, and the majority of the women were multiparous and had previously undergone a cesarean section (Table 2).

**Table 2:** Summary of reported cases of trophoblastic lesions arising in cesarean section scars (2006-2024)

Author	Year	Country	Number of patients	Age (Years)	Presentation	β-hCG levels (mIU/mL)	Diagnosis	Diagnostic tools used	Treatment provided	Outcomes and follow-up
Wu et al. [21]	2006	Taiwan	1	31	Persistent vaginal spotting	61798	Partial hydatidiform mole	TVUS, histopathology	Dilatation and suction curettage (D&C)	Vaginal bleeding for 9 weeks
Jin et al. [22]	2011	China	1	44	Irregular vaginal bleeding and lower abdominal pain	94724	partial mole	TVUS, histopathology	Suction and curettage	No evidence of disease
Ko et al. [23]	2012	Hong Kong	1	34	Persistent symptoms of pregnancy	219255	Partial hydatiform mole	TVUS, histopathology	Uterine artery embolization (UAE)	No evidence of disease
Chen et al. [24]	2013	Taiwan	1	41	Abnormal bleeding	1 (normal)	Coexisting ETT and PSTT from PSNs	USG, curettage, CT, histopathology	Total hysterectomy	No evidence of disease
Kaluarachchi et al. [1]	2013	Sri Lanka	1	40	Amenorrhea	6743	Complete hydatiform mole	Diagnostic laparoscopy	Subtotal hysterectomy	No evidence of disease
Qian and Zhu [6]	2014	China	1	22	Amenorrhea (47 days), irregular vaginal bleeding (15 days)	312468	CC	USG, histopathology	D&C, chemotherapy	No evidence of disease
Gómez García et al. [25]	2014	Spain	1	35	Routine checkup	1716	CSP	USG (Doppler scan)	Methotrexate (MTX), mifepristone	No evidence of disease
Zhou et al. [26]	2015	China	1	41	Lower abdominal pain, menstrual disorder	negative	APSN	USG, histopathology	Laparoscopy and removal of the lesion	No evidence of disease
Bekci et al. [27]	2016	Turkey	1	33	Increase β-hcG	146.762	GTD	USG, MRI	Selective UAE, total hysterectomy	Lung metastasis
Biswas et al. [28]	2016	India	1	31	Persistent nausea, post-abortion bleeding, enlarged uterus	15,000 → 31,332	PTD	TVS (Doppler), MRI	Chemotherapy (MTX + folinic acid)	No evidence of disease
Kulhan et al. [29]	2016	Turkey	1	20	Spotting vaginal bleeding	265	Placenta increta	TVS, Pathology	Total abdominal hysterectomy (TAH) (emergency)	No evidence of disease
Sherer et al. [30]	2016	USA	1	34	Mild pelvic pain and vaginal bleeding	76038	CC	USG, CT, histopathology	Laparotomy	Lung metastasis
Polat et al. [31]	2016	Turkey	1	42	Vaginal bleeding, abdominal pain	197,525 → 22,030 → 31,200	PTD	TVUS, histopathology	Suction and curettage, chemotherapy (MTX + folinic acid)	No evidence of disease
Hsiue et al. [32]	2017	Taiwan	1	54	Enlarging anterior abdominal mass around her cesarean section scar	8.3	GTD	CT, USG, incisional biopsy, Positron emission tomography (PET)	Chemotherapy (etoposide, MTX, actinomycin-D (ACTD), cyclophosphamide, vincristine (EMACO)), Wide excision of tumor	Recurrent tumor at the anterior abdominal wall and intestinal metastases
Zhang et al. [19]	2018	China	2	1. 28; 2. 29	1. Persistent amenorrhea; 2. Persistent amenorrhea	1. 132,660; 2. 114,725	1. Complete hydatidiform mole (GTN) 2. Complete hydatidiform mole (GTN)	1. B- USG; 2. B- USG, hysteroscopy	1. UAE, suction curettage, chemotherapy (MTX) 2. UAE, chemotherapy (MTX + ACTD)	No evidence of disease
Ling et al. [33]	2018	China	1	28	Irregular vaginal bleeding and mild abdominal pain	7894	Partial hydatidiform mole	TVUS, MRI, histopathology, Immunohistochemistry (IHC)	UAE	No evidence of disease
Nasiri et al. [34]	2018	Iran	2	1. 33; 2. 33	1. Irregular bleeding; 2. Irregular bleeding	1. 67 → 0.7; 2. 3,664 → 4,325	1. PSTT; 2. CC	1. TVUS (Doppler scan), IHC staining 2. TVUS/MRI, IHC staining	1. ACTD → TAH; 2. MTX (failed) → TAH, ACTD	No evidence of disease
Kwon et al. [35]	2019	Korea	3	1. 38; 2. 44 3. 40	1. Amenorrhea, vaginal spotting; 2. Amenorrhea, abnormal bleeding; 3. Amenorrhea, scant bleeding	1. not mentioned; 2. 450,945.9 → 11,427.2 → 16,180.9 → undetectable; 3. 2045.7 → normalized	1. CSP; 2. Partial hydatidiform mole; 3. Cervical pregnancy	1. USG, laparoscopy; 2. USG, laparoscopy; 3. USG, laparoscopy	1. Laparoscopic Transient occlusion of uterine artery (TOUA), excision; 2. D&C, TOUA, later laparoscopic excision + ACTD; 3. Failed D&C → MTX → laparoscopic TOUA	No evidence of disease
Zeng et al. [36]	2019	China	1	39	Asymptomatic abdominal mass at scar site	7.20 (recurrence)	Mixed GTN: 90% ETT + 10% CC	USG, MRI, IHC	Resections, hysterectomy	No evidence of disease
Gromis et al. [37]	2019	America	1	52	Persistent vaginal bleeding	453839	CC	TAUS, TVUS (Doppler scan), CT, MRI	Laparoscopic hysterectomy and bilateral prophylactic salpingectomy	No evidence of disease
McCarthy et al. [38]	2019	San Antonio	1	41	Chronic pelvic pain	negative	APSN	Histopathology	Hysterectomy	No evidence of disease
Yang et al. [39]	2020	China	1	39	Asymptomatic abdominal wall mass	<1.2 → 6.17 → 10.68	Mixed ETT (90%) + CC (10%)	PET, USG, CT/MRI, histopathology, IHC	Local excision, EP chemotherapy, hysterectomy	No evidence of disease
Liu et al. [40]	2020	China	1	35	Irregular vaginal bleeding	193,079 → 19,600	Hydatidiform mole (repetitive)	TVUS, pathology	UAE + MTX + suction evacuation	No metastasis
Zhou et al. [41]	2020	China	2	1. 32; 2. 34	1. Amenorrhea + bleeding, large mass; 2. Amenorrhea, vascular mass	1. >225,000; 2. 72,587 → 184.3	1. CC; 2. CSP	1. TVUS (Doppler scan), MRI; 2. TVUS, MRI	1. Embolization, chemotherapy, hysterectomy; 2. Embolization, curettage, laparoscopic resection	No evidence of disease
Lin et al. [8]	2021	Taiwan	2	1. 27; 2. 36	1. Intermittent vaginal spotting; 2. Persistent vaginal spotting	1. 98,199; 2. 34,098	1. CC; 2. Cervical hydatidiform mole	1. TAUS (Doppler scan), MRI; 2. TVUS, MRI	1. Uterine artery ligation (UAL), D&C; 2. LUAL, D&C.	No evidence of disease
Anicic et al. [42]	2021	Serbia	1	42	Vaginal bleeding	not mentioned	ETT	USG, histopathology	Surgery	No evidence of disease
Azimi et al. [43]	2021	Iran	1	41	Vaginal bleeding	1000	CC	USG	MTX, hysterectomy	No evidence of disease
Daggez and Dolanbay [44]	2021	Turkey	1	25	Amenorrhea (40 days), vaginal bleeding (3 days)	41.616	Molar CSP	TVUS, MRI, histopathology	Suction and curettage	No evidence of disease
Black et al. [45]	2021	Canada	1	36	Vaginal bleeding	negative	ETT	Excision and histopathology	Surgical repair of the cesarean scar defect	No evidence of disease
Qu et al. [46]	2022	China	1	32	Cessation of menstruation (68 days), Irregular vaginal bleeding (3 days)	265954→15,094	GTN	TVUS, MRI	Uterine evacuations, chemotherapy, and ablation	No evidence of disease
Huang et al. [10]	2023	China	2	1. 36; 2. 25	1. Mild uterine hemorrhage; 2. Uterine hemorrhage	1. 603; 2. 800	1. CSP; 2. CC	1. USG, MRI, CT; 2. USG, MRI, CT	1. Cesarean scar repair surgery by laparoscopy combined with hysteroscopy after UAE; 2. Chemotherapy	No evidence of disease
Tarafdari et al. [11]	2023	Iran	1	31	Delayed menses and spotting	3992	CC	TVUS	Hysterectomy	No evidence of disease
Al-Bataineh et al. [4]	2023	Jordan	1	37	Persistent vaginal discharge with streaks of blood and lower abdominal pain	43	Molar CSP	USG, diagnostic laparoscopy	Laparotomy	No evidence of disease
Hosseinmousa et al. [47]	2024	Iran	2	1. 34; 2. 40	1. Hypogastric pain and tenderness; 2. Vaginal spotting	1. 27633; 2. 225000	1. Invasive hydatidiform mole; 2. Invasive hydatidiform mole	1. TVUS (Doppler scan), histopathology; 2. TVUS (Doppler scan), histopathology	1. Suction, laparotomy; 2. Suction, hysterectomy, laparotomy, MTX	No evidence of disease
Chen et al. [48]	2024	China	1	38	Vaginal spotting	22,200 → 76,196 → 2,799 → 8.5	EPS (misdiagnosed as GTN)	TVUS, MRI, histopathology, IHC	Hysteroscopy, MTX, hysterectomy	No evidence of disease
Baekelandt et al. [49]	2024	Sweden	1	34	Amenorrhea, cesarean scar niche	Not specified (partial mole)	Partial molar pregnancy	TVS + Doppler	UAE + suction evacuation	No evidence of disease

Clinical Presentation

Amenorrhea, pelvic or lower abdominal pain, and irregular or continuous vaginal bleeding were the most often reported presenting symptoms. Additionally, a few individuals, particularly those with CC [6,37], had raised serum β-hCG levels, ranging from slightly elevated to extremely high values above 300,000 mIU/mL. However, APSNs and ETTs sometimes appear with normal [24,39] or low [26,38,45] β-hCG levels, making a prompt diagnosis challenging.

Type and Distribution of Lesions

The cases reviewed encompassed a wide spectrum of gestational trophoblastic and related lesions, reflecting the clinical and pathological diversity of these conditions. The most frequently reported diagnosis was hydatidiform mole [8,40,47], observed in both complete [1,19] and partial [21-23,49] forms, often with progression to PTD [28,31] or GTN [19,27,32,46]. These accounted for approximately one-third of all cases. CC [6,8,10,11,30,34,37,41], a malignant and highly vascular form of GTN, was the second most common lesion, reported either as a primary diagnosis or arising from prior molar pregnancies or cesarean scar sites. Several cases involved CSP [4,10,25,34,35,41,44] and CC, highlighting the increasing recognition of these entities in patients with prior uterine surgery. ETT [45] and PSTT [34], which are rare and often indolent forms of GTN, were documented in a small subset of patients, often presenting with lower β-hCG levels and requiring immunohistochemical confirmation. Unusual entities such as APSN [26,38] were also noted, usually associated with low or negative β-hCG and discovered incidentally or due to atypical bleeding. Notably, mixed histologies were reported in select cases, such as coexistence of ETT and PSTT [24] or combined ETT and CC [36,39], emphasizing the diagnostic complexity and the need for histological precision.

Diagnostic Approaches

The diagnostic workup across the reported cases employed a multimodal approach combining clinical assessment, imaging, histopathology, and, in selected instances, immunohistochemistry. TVUS/TAUS served as the primary imaging modalities, often enhanced with color Doppler [8,25,28,34,37,41,47,49] to assess vascularity in suspected GTD and CSP. MRI was frequently utilized as a complementary tool to delineate the extent of myometrial or extrauterine invasion, particularly in complex cases such as ETT, PSTT, or CSP-associated CC [8,10,27,28,33,41,44,46,48]. CT [10,24,30,32,37,39] and PET [32,39] scans were selectively employed for staging, especially in patients with suspected metastases or recurrent disease. 

Treatment and Outcomes

Management strategies were primarily surgical including suction curettage [8,19,21,22,31,35,40,44,47,49], hysterectomy (total or subtotal) [1,11,24,29,34,36-39,41,43,47], UAE [19,23,33,40,49], chemotherapy regimens [6,10,39,41,46](MTX [19,25,28,31,34,35,40,43,47,48], ACTD [19,34,35], EMACO [32]), and, in selected cases, laparoscopic or hysteroscopic resection [32,35-37,41]. Post-treatment outcomes were favorable in the vast majority of cases, with 58 patients achieving complete remission with no evidence of disease on follow-up. Two patients experienced lung metastases [27,30], and one case had local tumor recurrence and intestinal metastases despite combined chemotherapy and surgical excision [32]. Only a single case reported prolonged post-treatment vaginal bleeding, which ultimately resolved [21].

Discussion

Trophoblastic lesions occurring within cesarean section scars are an uncommon but clinically relevant subtype of GTD. The present systematic review synthesizes the clinical presentation, diagnostic process, treatment approach, and outcomes of these uncommon lesions from 36 well-documented case reports between 2006 and 2024.

Diagnosis of trophoblastic lesions in cesarean scars is especially difficult because they are rare and present nonspecifically. Typical symptoms, such as abnormal vaginal bleeding, amenorrhea, and lower abdominal pain, share commonality with other gynecologic conditions such as miscarriage, ectopic pregnancy, or even cesarean scar dehiscence. This makes frequent misdiagnoses likely, particularly that of CSP being confused with more virulent entities such as CC or ETT [48]. TVUS is the most commonly used first-line imaging modality. In several instances, Doppler examinations and MRI were required to further delineate vascularity, lesion size, and possible myometrial invasion [8,25,28]. Advanced imaging such as PET-CT was applied in some complex cases, especially for staging and assessment of suspected metastasis. For example, Hsiue et al. [32] employed PET-CT to identify metastases in a recurrent GTD patient, while Yang et al. [39] utilized PET-CT for assessment of a mixed ETT and CC. Both these examples highlight the significance of multimodal imaging in suspected cesarean scar GTD. Misclassification is not rare even with these developments. For instance, Chen et al. [48] reported a case of a condition that was initially diagnosed as GTN and subsequently proved to be exaggerated placental site (EPS), highlighting the imperative for histopathological diagnosis.

The lesions described in this review have a broad pathological spectrum. CC was the most common diagnosis (n = 8), followed by ETT (n = 5), invasive mole (n = 4), PSTT (n = 3), and APSNs (n = 2). The majority of the lesions were localized to the lower uterine segment or anterior uterine wall, locations which are associated with the cesarean scar. Interestingly, certain lesions like APSNs or ETTs had low or even within the normal range β-hCG levels, which made early diagnosis challenging [26,38,45]. On the other hand, CC most commonly had significantly high β-hCG levels, up to 450,000 mIU/mL in one instance [35,37].

The occurrence of molar pregnancies in a cesarean scar is rare. In the literature, just three cases have been reported. In 2006, Wu et al. [21] reported the first case. This patient had two cesarean procedures in the past and continued vaginal spotting after suction curettage. After the curettage material was examined pathologically, a partial molar pregnancy was identified. Three years later, a second case was documented by Michener and Dickinson [50]. The first thing they did was inject MTX into the gestational sac. But ten months later, the patient started bleeding profusely, which led to an emergency hysterectomy. Histopathological investigation revealed the presence of molar tissue during this procedure. A patient who had two previous cesarean deliveries and who continued to experience symptoms following a medical termination for a suspected partial molar pregnancy was the subject of the most recent case reported by Ko et al. [51] in 2012. To control the bleeding, UAE and suction evacuation were performed on her.

CC and other GTNs can metastasize, and in the cases considered here, two patients were reported to develop pulmonary [27] or intestinal metastases [32], thus supporting the importance of systemic assessment in high-risk presentations. Management plans were individualized based on lesion type, severity, and fertility preservation. Surgery was the pillar in all lesions. These comprised D&C, open or laparoscopic excision, UAE [19,51], and hysterectomy. For CC and other high-risk GTNs, systemic chemotherapy in the form of MTX-based regimens or EMA-CO [32] was administered in conjunction with surgical treatments [6,47]. Fertility-sparing strategies such as local resection and laparoscopic surgery were successful in several patients with limited disease [26,45,51]. The application of vNOTES, a conservative approach as in Baekelandt et al. [49], indicated a changing trend toward minimally invasive procedures in highly selected cases.

The majority of patients showed a positive outcome with no sign of disease in follow-up. This comprised patients who were treated conservatively as well as those on extensive procedures. Few cases presented with recurrence or metastasis, such as a patient with intestinal recurrence and a patient with pulmonary metastases [30]. Nonetheless, long-term data were not reported consistently. Largely because standardized follow-up times were lacking in most reports, there is a deficiency of post-treatment monitoring that underscores the necessity for standardized surveillance protocols, particularly for at-risk recurrence patients.

There are several limitations to this study. First, the evidence accumulated in this review comes exclusively from case reports and small case series, which have a lower level of evidence. These studies are more susceptible to publication bias since they tend to highlight unusual or successful cases without reporting unsuccessful or complex cases. Secondly, there was extensive heterogeneity in patient demographics, clinical presentations, diagnostic modalities, and treatment strategies among the included reports. This heterogeneity restricted the quantitative synthesis and the possibility of creating standardized management pathways. Thirdly, only published articles available in full text were considered, potentially excluding pertinent data that is either unpublished or not accessible.

## Conclusions

This systematic review emphasizes the intricate diagnostic and therapeutic profile of trophoblastic lesions in cesarean scars, highlighting the necessity for individualized, multidisciplinary management plans. Although good outcomes were reported in the majority of cases with proper treatment, the extreme variability in presentation and lack of standard protocols underscore the need for increased clinical awareness, enhanced diagnostic modalities, and collective data accrual to inform evidence-based practice in this rare yet potentially severe condition.
